# Release kinetics of VEGF_165 _from a collagen matrix and structural matrix changes in a circulation model

**DOI:** 10.1186/1746-160X-6-17

**Published:** 2010-07-19

**Authors:** Johannes Kleinheinz, Susanne Jung, Kai Wermker, Carsten Fischer, Ulrich Joos

**Affiliations:** 1 Department of Cranio-Maxillofacial Surgery, Research Unit "Vascular Biology of Oral, Structures (VABOS)", University Hospital Muenster, Waldeyerstrasse 30, D-48149, Muenster, Germany; 2 Private practice, Duelmen, Germany

## Abstract

**Background:**

Current approaches in bone regeneration combine osteoconductive scaffolds with bioactive cytokines like BMP or VEGF. The idea of our *in-vitro *trial was to apply VEGF_165 _in gradient concentrations to an equine collagen carrier and to study pharmacological and morphological characteristics of the complex in a circulation model.

**Methods:**

Release kinetics of VEGF_165 _complexed in different quantities in a collagen matrix were determined in a circulation model by quantifying protein concentration with ELISA over a period of 5 days. The structural changes of the collagen matrix were assessed with light microscopy, native scanning electron microscopy (SEM) as well as with immuno-gold-labelling technique in scanning and transmission electron microscopy (TEM).

**Results:**

We established a biological half-life for VEGF_165 _of 90 minutes. In a half-logarithmic presentation the VEGF_165 _release showed a linear declining gradient; the release kinetics were not depending on VEGF_165 _concentrations. After 12 hours VEGF release reached a plateau, after 48 hours VEGF_165 _was no longer detectable in the complexes charged with lower doses, but still measurable in the 80 μg sample. At the beginning of the study a smear layer was visible on the surface of the complex. After the wash out of the protein in the first days the natural structure of the collagen appeared and did not change over the test period.

**Conclusions:**

By defining the pharmacological and morphological profile of a cytokine collagen complex in a circulation model our data paves the way for further *in-vivo *studies where additional biological side effects will have to be considered. VEGF_165 _linked to collagen fibrils shows its improved stability in direct electron microscopic imaging as well as in prolonged release from the matrix. Our *in-vitro *trial substantiates the position of cytokine collagen complexes as innovative and effective treatment tools in regenerative medicine and and may initiate further clinical research.

## Background

### Osteogenesis

The human skeleton is subject to permanent remodelling processes: 5% of the human skeleton is rebuilt per year. This remodelling is an integral part also of the mechanism of bone healing and regeneration of bony defaults.

In the process of bone healing and regeneration, biochemical procedures follow a well-defined temporal and territorial pattern. Resting chondrocytes start to proliferate, differentiate into hypertrophic chondrocytes, and synthesise collagen and extracellular matrix.

Then blood vessels invade; osteogenesis takes place in the vicinity of neo-vessels that mediate the delivery of osteoprogenitors, secrete mitogen for osteoblasts, and transport nutrients and oxygen. The cartilage matrix is degraded and replaced with the typical trabecular bone matrix produced by osteoblasts. Blood vessels provide a conduit for the recruitment of cells involved in cartilage resorption and bone deposition and are therefore a crucial condition for any regeneration [1,2]. The process is operated by a variety of cytokines as bone morphogenetic proteins (BMPs) or vascular endothelial growth factor (VEGF) [3,4].

There are two basic options to support bone formation: to enhance the remodelling processes by optimizing the vascularization via application of potent angiogenetic cytokines as VEGF or to implant a scaffold to provide a matrix that induces bone regeneration [5,6].

### VEGF_165_

VEGF is an important cytokine in the process of endochondral bone development and mediating bone vascularisation for normal differentiation of chondrocytes and osteoblasts. An increase in VEGF is an indication of increased vascular permeability and microvascular activity, including angiogenic growth of new blood vessels [7-9].

VEGF is a homodimer glycoprotein, its family includes 6 related proteins; VEGF_165 _is most common and biologically active [10]. It is released by many cell populations as fibroblasts, monocytes, macrophages or lymphocytes [11]. The corresponding receptors belong to the tyrosine kinase family. VEGF_165 _induces angiogenesis on different levels: it acts as mitogen especially on endothelial cells, raises the vessel permeability and dilatation by releasing NO and has chemotactic impact on other growth promoting cell populations [12]. The most potent stimulus for VEGF_165 _synthesis is lack of oxygen. Under hypoxia an increase in VEGF_165 _mRNA was shown and, in addition, the RNA's half-life was extended. This effect is translated by the hypoxia sensitive transcription factor HIF1. The instantaneous angiogenetic effect of VEGF_165 _is the increase in vessel permeability and mitogenic stimulation of endothelial cells. According to its potential VEGF_165 _is also involved in pathophysiological processes like tumour growth; mainly in hypoxic tumour regions raised VEGF_165 _levels were scored [13,14]. Disadvantageous for a routine use are a difficult handling of the liquid application form, its short half-life and susceptibility to light and temperature.

### Bone graft substitutes and collagen

Some of the common methods used to repair bony skeletal defects are autografts, allografts, or synthetic implant materials. Yet, imperfections persist in these methods, such as limited harvesting, the possibility of disease transmission, poor biocompatibility, and the risk of prosthetic implantation failure. Therefore, alternative strategies, such as tissue engineering approaches, are needed to improve the treatment and quality of life of all patients.

The minimum requirements for bone graft substitutes are:

• No cancerogenic effect

• No water-solubility

• Non-immunogenic effect

• Lacking of an inflammatory response

• Defined bio-degradation and

• Biocompatibility, namely of the surface.

Widely-used materials are hydroxylapatite and tricalcium phosphate as synthetic inorganic bone graft substitutes. They come with good biocompatibility and osteoconductivity. Yet, they are brittle and not resilient in functionally stressed areas [15-17]. The advantage of collagen as a natural substitute is the fact that collagen is the main constituent of organic bone matrix. Fitted in bony defaults it is not degraded by but incorporated into the regenerating tissue. It accelerates the healing process and reduces the side effects of decomposition products [18,19].

In innovative approaches the osteoconductive collagenous scaffold is combined with the osteoinductive impact of cytokines like BMP or VEGF_165_. The objectives of our study were to apply VEGF_165 _in gradient concentrations to an equine collagen carrier and to study the complex in a circulation model. The VEGF_165 _release kinetics should be quantified and the morphological degradation of the collagen-cytokine complex should be visualized.

## Methods

### VEGF_165_-collagen complex

Collagen I was purchased (Resorba, Nuernberg, Germany) and liquefied. Human recombinant VEGF_165 _(R&D Systems, Wiesbaden, Germany) was added in different concentrations. The complexes were formed in hemispheres and drugged with aldehyde to avoid the cross-linking of collagen fibrils.

The total quantity of collagen was 5.6 mg/cm^3 ^per application, VEGF_165 _was added in 0.8 μg, 10 μg or 80 μg quantities.

### Circulation model

We used a digitally controlled peristaltic pump that delivered the medium with a mean flow rate of 27 ml per minute (Cole Parmer Masterlex Console Drive Pump). As aqueous solution a 0.2 mol PBS buffer was utilized in a total quantity of 80 ml. Circulation was simulated under constant conditions of 20°C and pH 7.2.

### Lab report

The complexes were charged with VEGF_165 _in three different concentrations: 0.8 μg, 10 μg and 80 μg. Three complexes of each concentration were incubated for 5 days. As a sample, the total volume of buffer medium was extracted and analysed to avoid saturation of the buffer medium with free VEGF_165_. To differentiate between the initial degradation of our collagen complexes with a quick VEGF_165 _release and the slow long-term saturation process, we adopted an asymmetrical test pattern:

On day one we took samples after 30 min, 1, 2, 4, 8, 12 and 24 hours. The next specimens were taken after day 2, 3, 4 and 5. VEGF_165_-free collagen complexes served as negative controls and were analysed identically.

### ELISA

VEGF_165 _concentrations were assessed by performing a solid-phase VEGF_165 _Immunoassay (VEGF_165 _Quantikine, DVE00, R&D Systems GmbH, Wiesbaden-Nordenstadt, Germany). The ELISA was performed according to the manufacturer's protocol; its sensitivity was described as < 9 pg/ml. The concentration of VEGF_165 _was expressed as pg/ml.

VEGF_165 _was quantified by using a standard curve made by human VEGF_165 _ranging from 31.2 pg/ml to 2000 pg/ml. The chromogenic reaction was read at 415 nm (Molecular Devices).

### Light microscopy

Collagen samples were processed according to a standard protocol. In short, they were fixed, dehydrated in increasing gradients of ethanol and embedded in paraffin. Thin sections were sliced, stained according to an azan standard procedure and fixed in methacrylate.

The sections were evaluated with a light microscope (Zeiss Axioscop, Jena, Germany).

### Scanning electron microscopy (SEM)

Samples were fixed in 3% glutaraldehyde in 0.1 mol phosphate buffered saline and then washed in the buffer (0.1 mol PBS). After rinsing, the samples were dehydrated in a graded ethanol series and dried with a critical point drying. All dried samples were mounted on aluminium stubs and sputter coated with coal to a coating thickness of 8 nm.

For immunohistochemical SEM analysis the sections were fixed in 4% paraformaldehyde solution, rinsed with 0.1 mol PBS buffer and incubated with primary VEGF_165_-specific antibodies at room temperature for 1 hour. Afterwards, the secondary immunogold-labelled antibody was incubated at room temperature for 1 hour. Between incubation steps phosphate buffered saline rinses were performed. All antibodies were diluted according to the manufacturers' instructions.

The gold particles as spheres of a 10 nm diameter were easily detectable in scanning electron microscopy.

### Transmission electron microscopy (TEM)

For TEM analysis the collagen samples were fixed in 3% glutaraldehyde for 24 hours, rinsed in 0.1 mol phosphate buffered saline and incubated in osmium acid for 1 hour.

Afterwards, the samples were dehydrated in a graded ethanol series, embedded in araldite and sliced thin sections (1 μm). The slices were stained with tolouidin blue following a standard procedure. Representative areas were cut in ultra-thin slices of 70 nm, placed on copper nets and analysed in transmission electron microscopy.

Immunohistochemical staining was performed as described before; the gold spheres in TEM presented as dark areas.

## Results

### VEGF_165 _half-life

**Figure 1:**
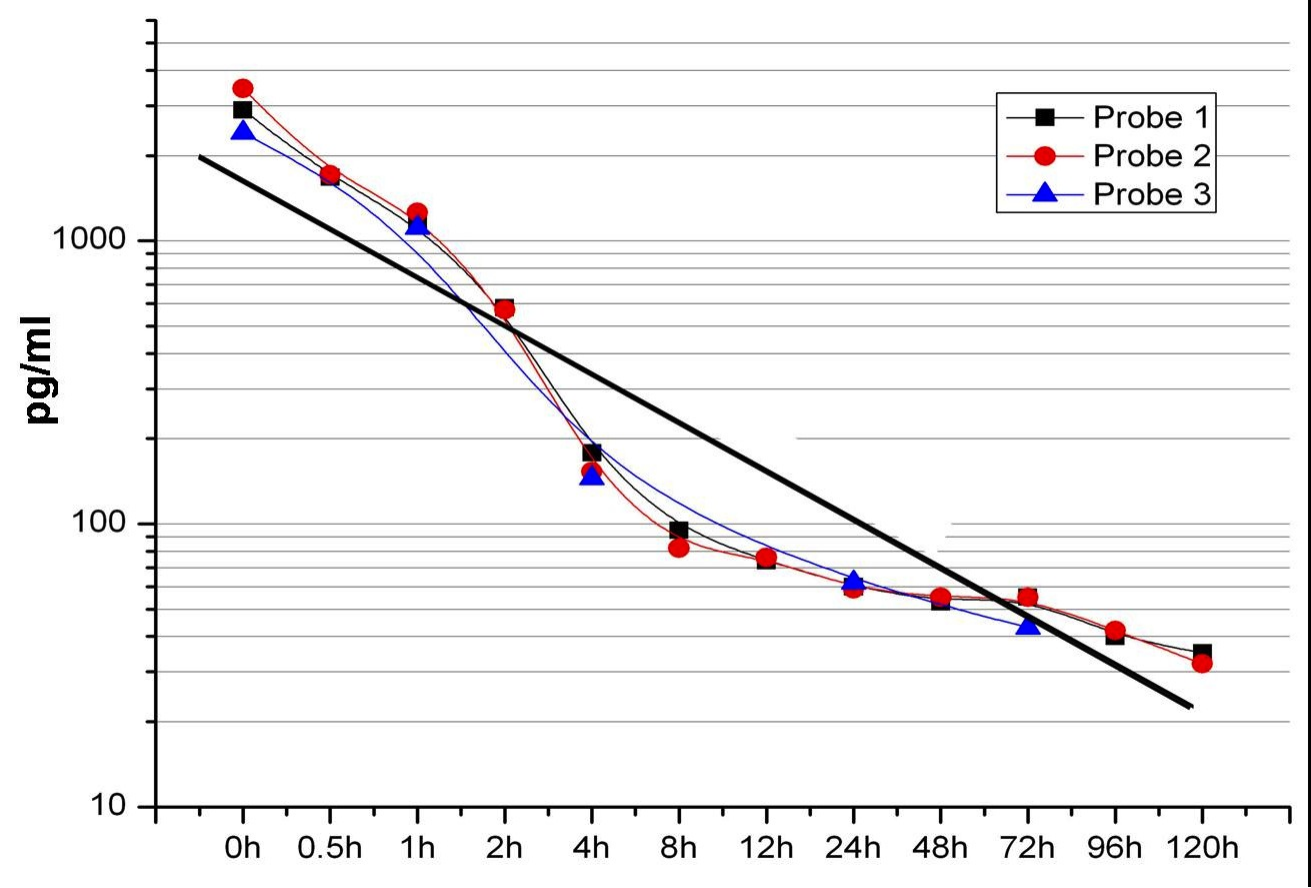
**Half-life of VEGF**.

To determine biological half-life of VEGF_165 _its dissolution in aqueous solution at room temperature was analysed. VEGF_165 _collagen complexes charged with 10 μg of VEGF_165 _were probed over 12 hours. Our results provide a half-life of free VEGF_165 _of 90 minutes (Fig. 1).

### VEGF_165 _release kinetics

**Figure 2:**
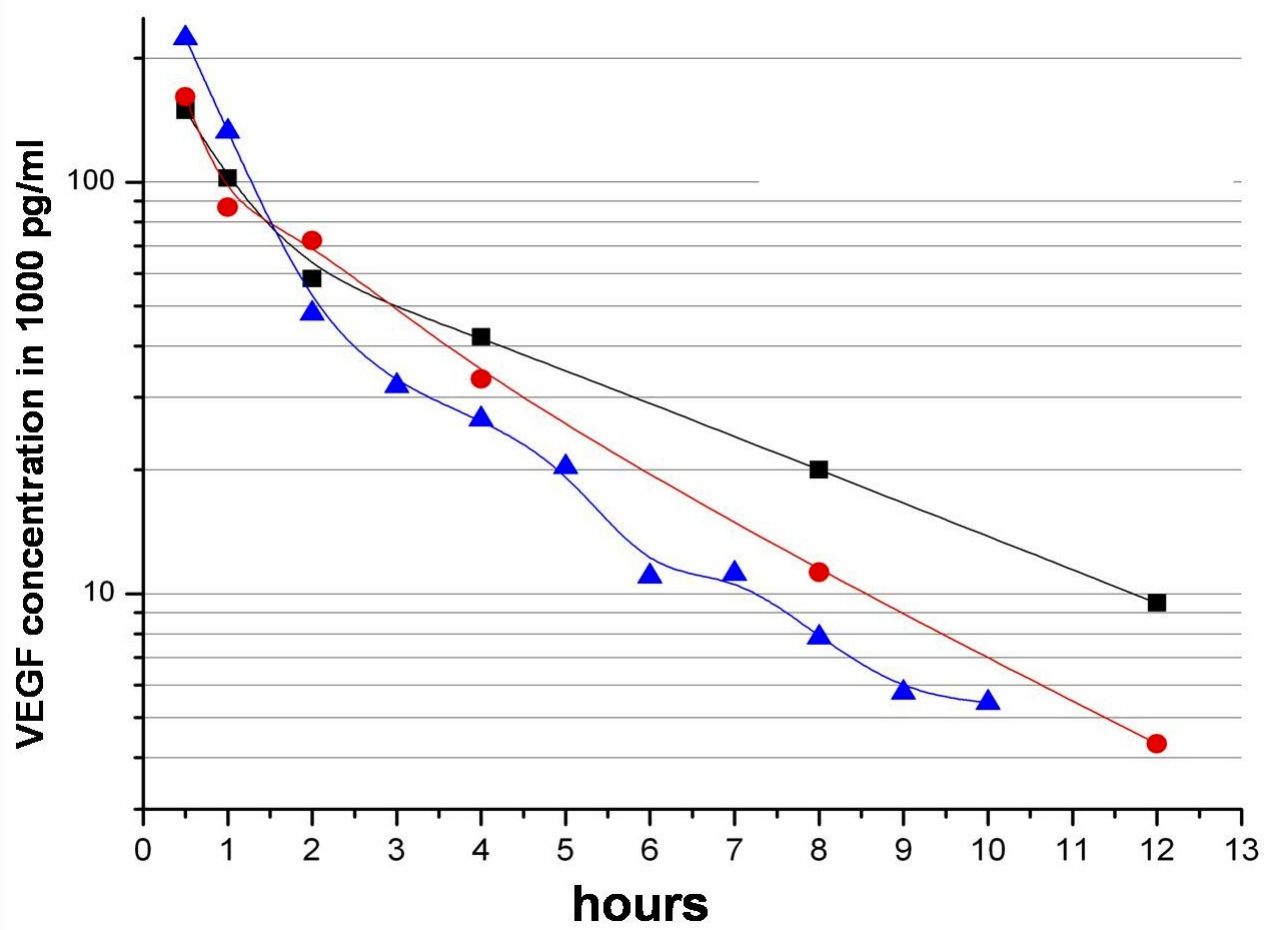
**Release kinetics of VEGF**.

In a half-logarithmic presentation the observed VEGF_165 _concentration showed a characteristic linear decline over time. The gradients of the three VEGF_165 _doses were parallel and independent of VEGF_165 _concentration. VEGF_165 _release reached a plateau after 12 hours and was no longer detectable in the applications of 0.8 μg and 10 μg after 48 hours, whereas the complex charged with 80 μg of VEGF_165 _still showed measurable cytokine release after over 50 hours. Saturation effects of the buffer medium were not observed (Fig. 2).

### VEGF_165 _degradation

**Figure 3:**
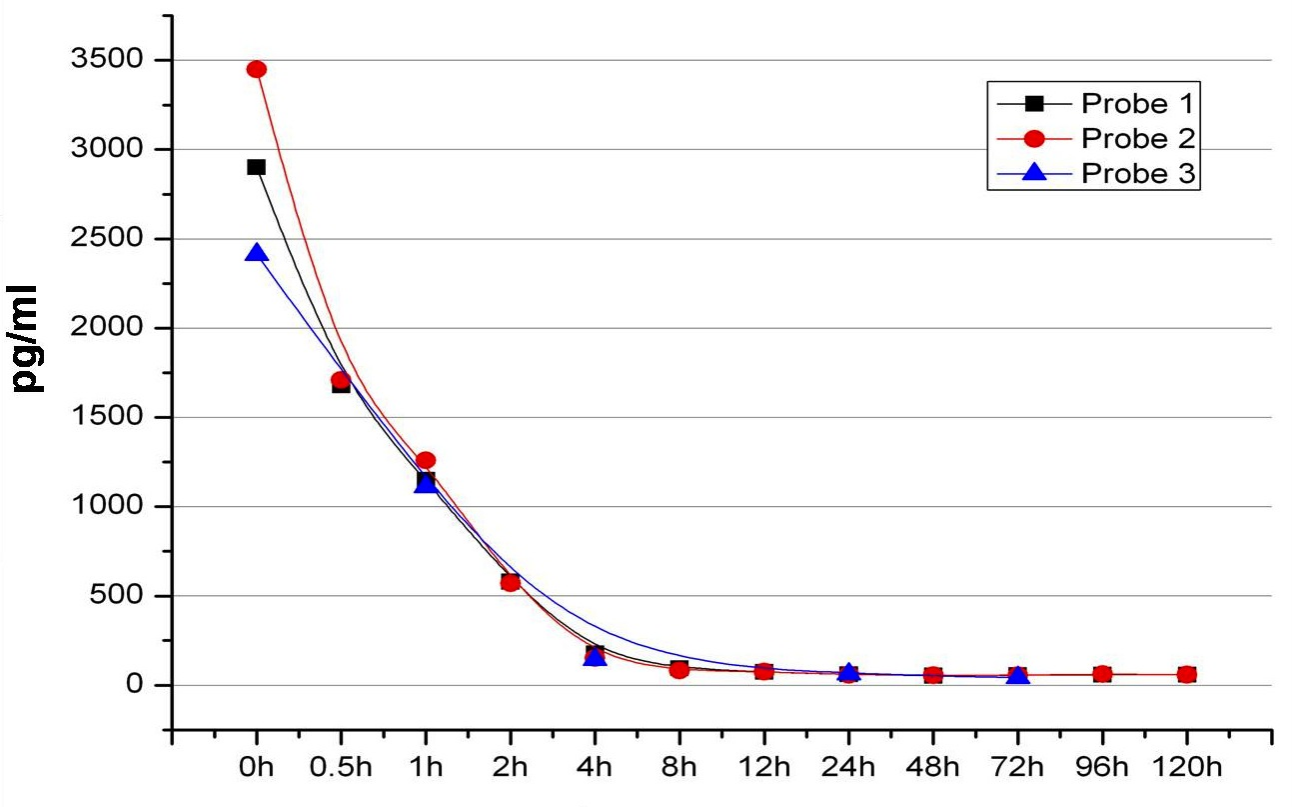
**Natural degradation of VEGF**.

The efficiency describes the quotient of VEGF_165 _values scored in our test setting and initially applied VEGF_165_. Only 10% of initially applied 0.8 μg were finally detected in the present study. Ninety per cent were lost during production, transport or storage. Of the applied 10 μg and 80 μg, 96% respectively 97% were lost (Fig. 3).

### Light microscopy

**Figure 4:**
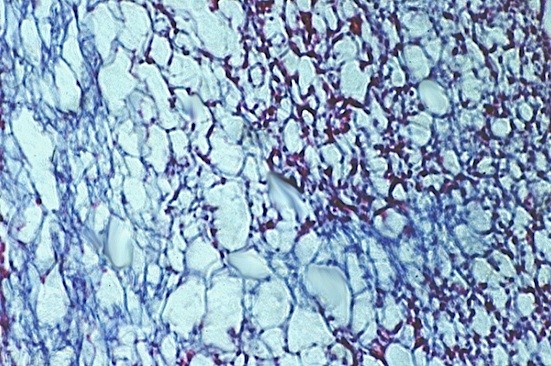
**Collagen matrix, azan staining (100×): representative central area of pure collagen matrix**.

In light microscopy the VEGF_165 _collagen complex appears homogenously, presents a reticular structure and shows no signs of structural defaults caused by fixation or coupling with VEGF_165_. Only in the periphery single agglutinated fibres are detected; these are artefacts caused by the production process (Fig. 4).

### SEM

**Figure 5:**
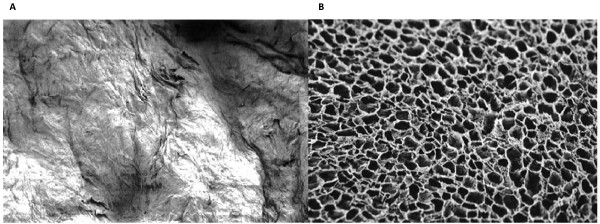
**Collagen matrix with (a) and without (b) VEGF, SEM (100×); the smear layer coffering the surface of the collagen matrix can be seen on the left picture**.

In scanning electron microscopy the VEGF_165 _collagen complexes feature more agglutinated parts, even in central areas, in contrast to the collagen matrix without cytokine (Fig. 5a and 5b).

**Figure 6:**
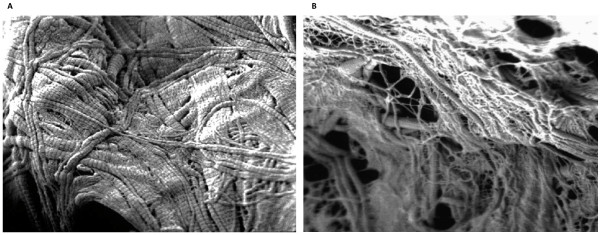
**VEGF_165_-collagen complex on day 3 (a) and day 5 (b), SEM (20000×)**.

During the five days of degradation process the ultra structure of the VEGF_165 _collagen complexes changes considerably. On day 0, the collagen matrix is coated by a VEGF_165 _layer that varnishes the single collagen fibrils. After 3 days of simulated circulation the collagen fibres are clearly detectable; this effect is more obvious on day five. The collagen matrix appears porose and knotty (Fig. 6a and 6b).

**Figure 7:**
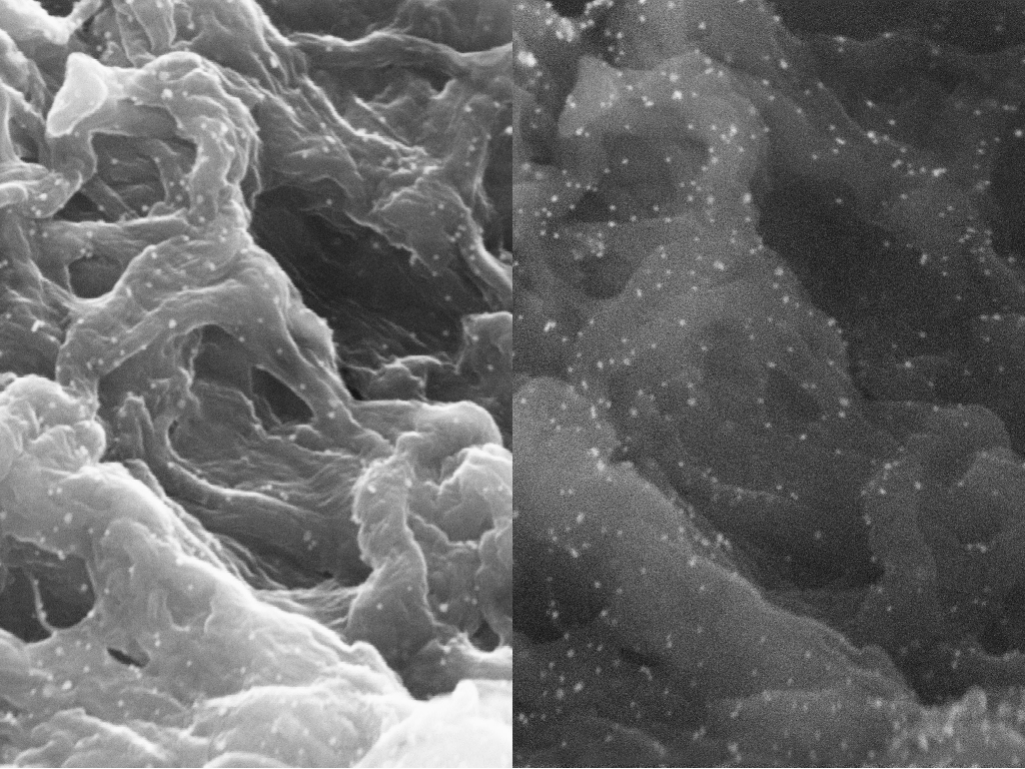
**VEGF_165_-collagen complex, 10 μg, TEM, (5000×)**.

With immuno-gold-labelling the VEGF_165 _molecules are visible. A homogenous distribution of VEGF_165 _in the collagen scaffold can be proved (Fig. 7).

### TEM

**Figure 8:**
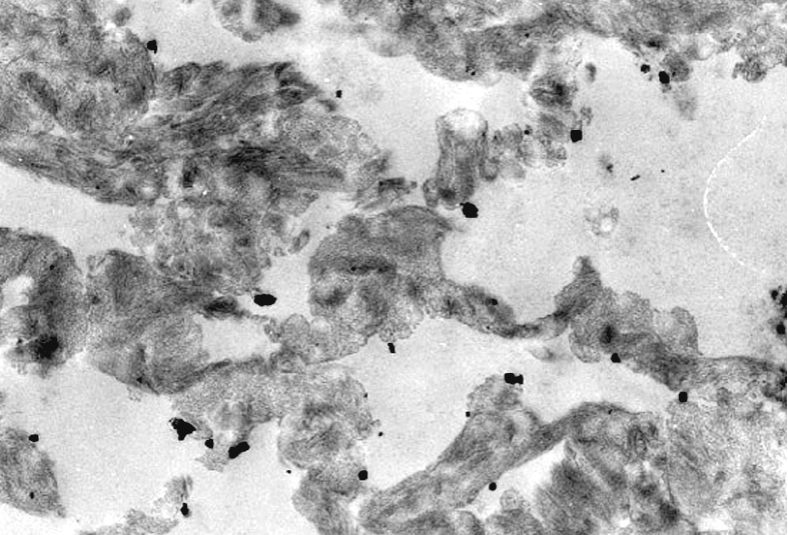
**VEGF_165_-collagen complex, 10 μg, TEM (3400×)**.

**Figure 9:**
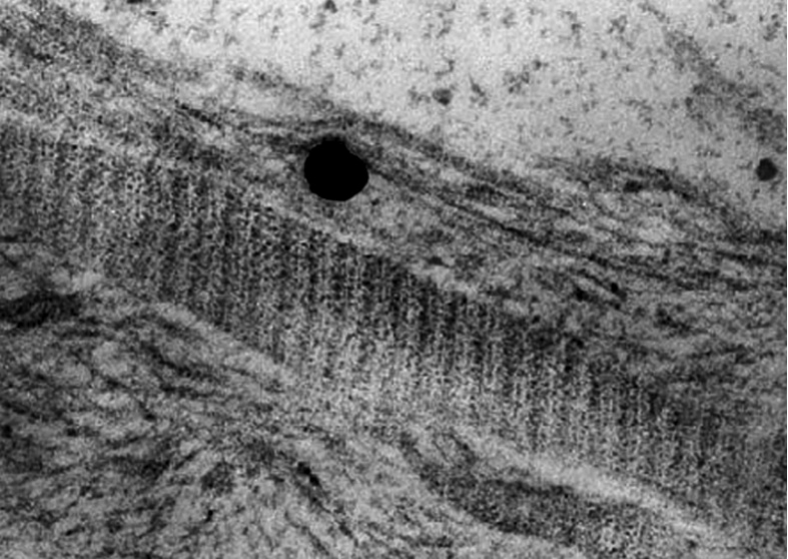
**VEGF_165_-collagen complex, 10 μg, TEM, (21500×); a VEGF-antibody complex in relation to its collagen fibre**.

In transmission electron microscopy the gold particles present themselves as black round structures (Fig. 8). Single VEGF antibody complexes can be precisely assigned to their corresponding collagen fibril. Due to the close vicinity between fibre and VEGF an adhesion must be assumed that overcomes the preliminary chemical procedure for TEM (Fig. 9).

## Discussion

To restore form and function to an existing bony defect, vascularisation is the key to success.

Clinical experience shows that avascular bony structures namely in chronically infected bones tend to atrophy and fracture [20].

Circulation and angiogenesis are responsible for a restored perfusion of impaired bone areas.

Bone cells on the other hand release growth factors to stimulate angiogenesis. Osteo- and angiogenesis are clearly linked in a strong co-dependent relation. The high susceptibility and the low applicable doses of cytokines make high demands: next to good biocompatibility, an easy application mode is critical for the successful use of biomaterials for regenerative medicine strategies [21,22]. VEGF_165 _has been exposed as the central angiogenetic protein in the process of bone regeneration; many *in-vitro *studies underlined its potency to stimulate osteogenesis physiologically via induction of neo-vascularisation [23]. Xenogenic collagen is a well established drug carrier in daily clinical use. As freeze-dried sponge it comes with excellent biocompatibility and is hence the ideal carrier for cytokine application.

In the present study the combination of a xenogenic collagen carrier and recombinant human VEGF_165 _is analysed pharmacologically and morphologically. This kind of research is crucial for forthcoming *in-vivo *studies where biological factors will overlie and falsify the therapeutical effects of the VEGF_165 _collagen complex. To be able to interpret these results properly drug release kinetics has to be established before. In cell cultures the VEGF_165 _specific half-maximum growth stimulation has been determined. The effect of applied cytokines is supposed to range above this score [24].

Our data accounts for VEGF_165 _release from the collagen over 48 hours; considering the 90 minutes half-life of free VEGF_165 _it is a surprising result. Obviously, a stabilisation of VEGF_165 _can be achieved by connecting the cytokine with collagen fibrils. The trial at hand provides only indirect evidence for this assumption but is observed in the whole test series.

During the first 50 hours an elevated release rate was observed as described in the literature before. The VEGF_165 _release is divided in two phases: first, the quick elusion of VEGF_165 _and diffusion into the buffer medium, and second, the slow sustained disposal when the VEGF_165 _molecules are dissolved from the degrading collagen fibrils in the deeper areas of the matrix.

This pharmacological behaviour corresponds with our morphological findings in REM: hydrolytic erosion reveals the single collagen fibrils and facilitates VEGF_165 _release.

The fraction of released VEGF_165 _varies in our data from 3% to 10%. Despite ideal test condition the main section of VEGF_165 _is lost during production, transport and storage.

The decreasing efficacy of the higher concentrated VEGF_165 _carriers argues for a saturation effect, higher doses of VEGF_165 _in the collagen scaffold do not lead to higher VEGF_165 _release [6].

To sum up: The biphasic release kinetic allows a hyperphysiological stimulation caused by the applied VEGF_165 _over 50 hours. It is more efficient than free VEGF_165_. Higher doses of VEGF_165 _do not lead to better effects for there is no proportional connection between the dose in the collagen carrier and the emitted total quantity.

The next steps to elucidate the biological behaviour of the cytokine collagen complex are *in-vivo *trials to eliminate the shortcomings of our setting

- PBS as an inadequate model for blood flow in human tissues

- disregard of enzymatic degradation processes

- insufficient verification of biologically active cytokine areas

The interfacing of VEGF_165 _to a collagen scaffold is not the only way of cytokine application: its transport in micro spheres was described; cytokine mRNA was coupled with a viral vector and cytokine plasmid DNA was directly transferred into the tissue [25-27].

## Conclusions

The restitution of bony defaults with a technique that provides biologic functionality, easy mechanical handling and reliable outcome is a significant challenge in maxillofacial surgery.

Our idea was to combine an osteoconductive scaffold with osteoinductive proteins and hence to stimulate and support natural healing and regenerating processes.

Our *in-vitro *trial substantiates the position of cytokine collagen complexes as innovative and effective treatment tools in regenerative medicine and paves the way for further clinical research.

## Competing interests

The authors declare that they have no competing interests.

## Authors' contributions

CF established the circulation model.

JK carried out the immunoassays.

SJ and KW participated in the design of the study and performed the statistical analysis.

UJ, JK and CF conceived of the study, and participated in its design and coordination and helped to draft the manuscript.

CF and UJ were involved in revising the article.

All authors read and approved the final manuscript.
